# Analysis of Visual Risk Factors of Anterior Cruciate Ligament Injury of Knee Joint

**DOI:** 10.3390/jcm11195602

**Published:** 2022-09-23

**Authors:** Zhong Chen, Yuheng Li, Yichi Zhang, Zhengzheng Zhang, Jingsong Wang, Xinghao Deng, Chengxiao Liu, Na Chen, Chuan Jiang, Weiping Li, Bin Song

**Affiliations:** 1 Department of Sports Medicine, Sun Yat-sen Memorial Hospital, No.107 on Yanjiang Road West, Guangzhou 510120, China; 2 Department of Trauma Orthopedics, Chongqing Ninth People’s Hospital, Chongqing 400700, China; 3 Department of Ophthalmology, Sun Yat-sen Memorial Hospital, No.107 on Yanjiang Road West, Guangzhou 510120, China; 4 Clinical Research Center, Sun Yat-sen Memorial Hospital, No.107 on Yanjiang Road West, Guangzhou 510120, China

**Keywords:** anterior cruciate ligament, sports injury, visual function, risk factors

## Abstract

This study aimed to explore whether the defect of visual function is a risk factor of knee anterior cruciate ligament (ACL) sports injury and to provide a theoretical basis for the primary prevention of ACL sports injury. This cross-sectional study included 392 participants divided into two groups: the sports injury group (287 with sports injury of knee) and the control group (105 healthy volunteers). Participants in the sports injury group were further divided into the ACL-Intact group (133) and the ACL-Deficient group (154). Participants in the sports injury group received a questionnaire about the conditions of their injury (including injury action, site condition, weather, contact) and a visual examination by synoptophore (including binocular vision, subjective and objective oblique angle, visual fusion range, stereoacuity). Participants in the control group only received the visual examination. In the end, we found that low visual fusion range (*p* = 0.003) and injury action, especially quick turn (*p* = 0.001), sudden stop (*p* < 0.001) and jump (*p* = 0.001), are the major risk factors for ACL injury in the analysis of the integrated data. In addition, athletes with low vision fusion range have increased risk of ACL sports injury when they make a sudden stop on wooden floor, plastic floor or cement floor on cloudy days (OR = 13.208). Visual factors, especially low fusion range, significantly increase the risk of ACL sports injury.

## 1. Introduction

Anterior cruciate ligament (ACL) injury of the knee joint is one of the most common sports injuries for athletes, accounting for nearly 50% of knee joint injuries, which often needs surgical treatment [1,2]. ACL injury causes a series of short-term and long-term adverse effects. The short-term consequences of ACL injury include ligament reconstruction surgery, long-term rehabilitation training, suspension of previous sports, early termination of sports careers and so on. In the long run, whether ACL reconstruction surgery is performed or not, the risk of knee osteoarthritis (OA) and lifelong disability increases significantly. Osteoarthritis secondary to ACL and meniscus injury has brought a huge economic burden to society [3]. Therefore, the prevention of ACL injury is an important goal of public health intervention. In a sense, the research on the prevention of ACL injury is more socially significance than the research on the treatment of ACL injury [4]. Therefore, in order to reduce the incidence of ACL injury in sports, we first need to explore the risk factors of ACL injury.

Current research suggests that the risk factors of ACL can be roughly divided into internal factors and external factors. Among them, internal factors include demographic, neuromuscular, joint relaxation and bone morphological risk factors [5]. External factors are mainly the physical condition of athletes, including environmental factors and sports factors. At the actual injury scene, internal factors and external factors do not exist in isolation and often act together on the athletes. When the athletes themselves have some internal risk factors, the external risk factors in the sports environment further increase the risk of injury in the process of playing the sports.

At the same time, motor balance function is also an important factor [6,7]. Recent studies have shown that visual feedback is essential to determine balance, and the postural stability of individuals with low vision is worse than that with normal vision [8]. The influence of dim light can also cause visual changes in gait speed, playing football and trunk control parameters. Additionally, visual problems can lead to a more cautious and unstable gait [9]. On the other hand, researchers have found that stability in basketball players and other dynamic sports can be enhanced through visual training [10]. The above research shows that gait, balance function and sensory motor response time may be affected by visual function, which is closely related to the risk of sports injury. Therefore, we believe that abnormal visual function may be one of the important risk factors of ACL injury. However, through the search of literature libraries at home and abroad, we did not find any previous literature on the correlation between visual defects and ACL injury risk.

The purpose of this study was to explore whether the defects of visual-related functions are risk factors that lead to or increase the sports injury of the ACL.

## 2. Materials and Methods

### 2.1. Statement of Informed Consent and Ethical Board Approval

This study was approved by the medical ethics committee of Sun Yat-sen University (sysec-ky-ks-030) and registered in the China Clinical Research Registry (chictr180015581). 

### 2.2. Study Design and Data Collection

This study included patients with a knee sports injury who underwent knee arthroscopy in our hospital between January 2020 and January 2021. Inclusion criteria for the ACL-D group included patients with a knee sports injury in our department who had a clear history of sports injury and arthroscopic indications and an average exercise time per week in the year before injury of 1 to 10 h. Inclusion criteria for the ACL-I group included imaging data, physical examination and intraoperative arthroscopy which confirmed the existence of meniscus injury and indications of meniscus suture or plastic surgery without ACL injury; other aspects are the same as above. Inclusion criteria for the control group included no previous history of knee fracture or ligament, cartilage and meniscus injury; other aspects are the same as above. Patients with previous history of fractures, ligament, cartilage and meniscus injuries or operation on the knee were excluded. 

In this study, a questionnaire survey was used to explore the internal and external risk factors affecting knee sports injury. After signing the informed consent form, participants were asked to fill in questionnaires related to gender, age, weight, height, dominant side, injury behavior, site conditions, weather, contact condition and repeated injuries. These also included basic information about binocular vision and eye diseases.

**Figure A1:**
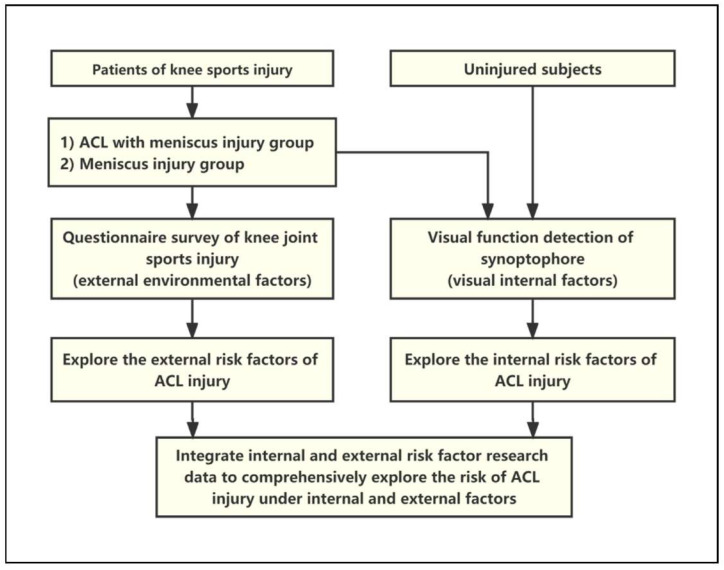
**Flow chart 2:** Research design and data analysis process.

Then, in order to further explore the correlation between visual factors and ACL injury, two experienced ophthalmologists used a synoptophore to check the visual function according to the operation specifications, including dominant eye, pupil distance, binocular vision, subjective and objective oblique angle of view, fusion range and stereo visual sensitivity. This study also included participants without sports injury as the control group to reduce the statistical deviation. The data of visual examination and external factors were used for comprehensive analysis to further confirm whether the combination of visual factors and external factors increases the risk of ACL injury (Figure A1).

### 2.3. Statistical Analysis

Sample size calculation: the sample size estimation of this study was completed by using software (SAS 9.4 statistical software, NCSU, USA) and the sample size calculation formula of multi-group medical record control studies. We set the α level to 0.05, and the test efficiency was 0.80. The standard deviation of the data was estimated and calculated. The minimum sample size of each group was 94 participants.

Analysis of questionnaire survey results: in order to explore which external factors increase the risk of ACL injury, we used the chi-square test or *t*-test of two independent samples (SPSS 25.0 statistical software, IBM, USA) to compare the differences between the meniscus injury group and ACL combined with meniscus injury group. Single-factor logistic regression analysis (r-4.0.1) was used as the primary screening with *p* < 0.2 as the standard. The items that met the primary screening criteria of single-factor logistic regression analysis were continued to carry out multi-factor logistic regression analysis (r-4.0.1) and further explore the environmental external risk factors of ACL injury with *p* < 0.05 as the standard.

Statistical analysis of visual examination results: in order to explore the visual risk factors of ACL injury, the chi-square test or analysis of variance (ANOVA) (SPSS 25.0 statistical software, IBM, USA) was used to compare the differences among meniscus injury group, ACL combined with meniscus injury group and uninjured control group. Univariate and multivariate logistic regression analysis (r-4.0.1) were used to explore the visual internal risk factors of ACL injury.

Comprehensive analysis of integrated data: in order to explore the risk of ACL injury under internal and external factors, we established a multi-factor logistic regression model to obtain the corrected OR value of each item, in which the OR value of each item approximately reflected the risk of ACL injury. The product of OR values approximately reflected the ACL damage risk when they existed together.

## 3. Results

### 3.1. Eye Disease Is a Major Risk Factor of ACL Injury from Questionnaires

A total of 287 patients were included in this part of the study, of which 133 patients had an ACL injury (ACL-D group, 46.34%), and 154 patients had no ACL injury (ACL-I group, 53.66%). The majority of participants in both groups were male; the average age was 29.14 ± 0.88 (years old). There was no significant difference between the two groups among age (*p* = 0.288), gender (*p* = 0.095), BMI (*p* = 0.222) and dominant side (*p* = 0.976) by *t*-test or analysis of variance test of the independent samples of the two groups (*p* > 0.05) (Table 1).

The results of the questionnaires showed that, in the ACL-D group, 72.18% of them were non-contact injuries, 63.16% on the dominant side, and 66.17% were single injuries. Regarding injury action, quick turn had the highest proportion, 24.06%; sunny day made up the highest proportion of weather factors (81.95%); and, in the site factors, the cement floor accounted for the most (38.59%). 

In terms of visual-related factors, 31.58% (ACL-D) and 37.01% (ACL-I) of the two groups reported that their binocular vision was normal, while other participants had different degrees of ametropia. In addition, most of the participants had no other eye diseases. Among the participants with eye diseases, the proportion of self-reported astigmatism was the highest, accounting for 30.08% (ACL-D) and 37.66% (ACL-I), respectively (Table 1).

In the chi-square test and *t*-test of the two groups of independent samples, as shown in Table 1, we found that the injury action (*p* = 0.010) was statistically significant between the two groups (*p* < 0.05). Although there were differences between the two groups in terms of contact condition (*p* = 0.702), site conditions (*p* = 0.125), weather (*p* = 0.915), right eye vision (*p* = 0.389), left eye vision (*p* = 0.391), history of eye diseases (*p* = 0.069) and multiple injuries (*p* = 0.312), they were found to be statistically significant (*p* > 0.05) (Table 1).

In the logistic regression analysis, we screened out the possible risk factors related to ACL injury according to *p* < 0.2 in the univariate logistic regression analysis. Among them, age (*p* = 0.129), gender (*p* = 0.096), weight (*p* = 0.033), BMI (*p* = 0.055), eye disease (*p* = 0.095), injury behavior (*p* = 0.014) and site conditions (*p* = 0.141) met the test criteria of *p* < 0.2 (Table 2). Then, seven items with a *p*-value of less than 0.2 in univariate analysis were included in the multivariate logistic regression analysis. Through the analysis, it was found that strabismus (*p* = 0.035) (eye disease) and quick turn (*p* = 0.008) (injury action) were significantly correlated with ACL injury (Table 3). These results suggest that rapid redirection (injury action) and strabismus (eye disease) are risk factors for ACL injury.

### 3.2. Low Visual Fusion Range Is a Major Risk Factor for ACL Injury from Visual Examination

In this study, a total of 377 people underwent visual function examination; 127 (38.46%) were in the ACL-D group, and 145 (33.69%) were in the ACL-I group. 

The participants of the visual examination were all participants that were included in the questionnaire. A total of 154 people in the ACL-D group and 133 people in the ACL-I group were enrolled. Nine people in the ACL-D group and six people in the ACL-I group, a total of 15 people, were lost. The control group included 105 uninjured healthy volunteers (27.85%). There were more male participants in the three groups. The average ages of the three groups were 28.72 ± 8.81 (ACL-I group), 27.71 ± 8.23 (ACL-D group) and 27.61 ± 7.99 (control group) years old (Table 4). 

In the ACL-I, ACL-D and control groups, we found that the main visual eye of most participants was the right eye, accounting for 83.46%, 85.52% and 83.81%, and the pupil distance was 62.06 ± 2.71 mm, 62.31 ± 2.76 mm and 63.23 ± 10.02 mm, respectively. Among the three groups, only about 45% of the participants had normal visual acuity on both sides, and most of the participants had different degrees of ametropia on at least one side.

The difference between subjective and objective squint angles can reflect the first level of stereo visual function. The larger the value, the higher the possibility of a squint. The proportion of strabismus (difference ≥ 5) in the ACL-D group (15.86%) was slightly higher than that in the ACL-I group (9.45%) and control group (9.52%). Compared with the mean value of the subjective and objective oblique angle difference between the three groups, the control group (1.69 ± 1.88°) was slightly lower than the ACL combined with meniscus injury group (1.97 ± 1.91°) and meniscus injury group (1.99 ± 2.19°).

The visual fusion range can reflect the second level of stereo visual function. The higher the value, the better the stereoscopic function. The proportion of people with abnormal visual fusion range (fusion range < 25°) in the ACL-D group (80.00%) was slightly higher than that in the ACL-I group (70.08%) and control group (71.43%). Compared with the average range of visual fusion among the three groups, the ACL-D group (17.82 ± 6.17°) was slightly lower than that of ACL-I group (20.26 ± 7.27°) and control group (22.07 ± 5.16°).

Stereoacuity can reflect the third level of visual function. The higher the value, the worse the stereoacuity. In the ACL-D group (66.90%) and ACL-I group (68.50%), the proportion of people with normal stereoacuity (stereoacuity ≤ 60″) was slightly lower than that in the control group (74.29%). The mean stereoacuity of the ACL-D group (273.93 ± 522.72″) was slightly higher than that of the ACL-I group (221.89 ± 411.15″) and control group (176.76 ± 361.42″).

The chi-square test and analysis of variance showed that the visual fusion range of the three groups was significantly different (*p* < 0.001), while gender (*p* = 0.494), age (*p* = 0.512), dominant eye (*p* = 0.883), pupil distance (*p* = 0.280), right visual acuity (*p* = 0.966) and left visual acuity (*p* = 0.922) were not. Meanwhile, there was no significant difference in oblique angle difference (*p* = 0.464) and stereopsis sensitivity (*p* = 0.233) (Table 4).

In the logistic regression analysis (Table 5 and Table 6), through the preliminary screening of single-factor logistic regression analysis, only the visual fusion range met the screening criteria of a *p*-value less than 0.2. Further, the multivariate logistic regression analysis results showed that only the visual fusion range was correlated with ACL injury when comparing the meniscus injury group (*p* < 0.001) and the control group (*p* < 0.001). These results suggest that low vision fusion range is the most likely internal risk factor for ACL injury.

### 3.3. Low Visual Fusion Range Combined with Extrinsic Factors Increases the Risk of ACL Injury

ACL injury is caused by internal and external factors. Therefore, in order to evaluate the risk factors of ACL injury, we conducted a comprehensive analysis of the visual fusion range and external factors of injury, including site conditions, weather and action. Based on the analysis results of visual function examination data, we believe that the fusion range is the most likely risk factor affecting the increase of ACL injury risk. Therefore, we replaced the column of “eye disease” in the questionnaire data with the data of fusion range and conducted logistic regression analysis again. The results showed that visual fusion range (*p* = 0.003) and injury action, especially quick turn (*p* = 0.001), sudden stop (*p* < 0.001) and jump (*p* = 0.001), were independent risk factors for ACL injury (Table 7).

Then, through the establishment of a multi-factor logistic regression model, we corrected the OR values of different items so that the product of their OR values reflected the risk of different factors at the same time. We ranked and superimposed the OR value of the visual fusion range with the first three items with the largest OR value among the external factors, such as site conditions, weather and action of injury. The results showed that the defect of visual fusion range was closely related to contact ACL injury (OR = 10.583) and non-contact ACL injury (OR = 13.208) (Table 8 and Table 9). This indicates that athletes with vision fusion range defects may have an increased risk of ACL non-contact sports injury when they make a sudden stop on wooden floor, plastic floor or cement floor on cloudy days.

## 4. Discussion

Through the comprehensive analysis of the external environmental factors of ACL injury collected by the questionnaire and the internal visual factors collected by visual inspection, this study found that athletes with abnormal visual fusion range have increased risk of ACL non-contact and contact injury when they perform a quick turn, sudden stop, jump or other actions on hard ground, such as wooden floor or cement ground, on cloudy days.

In this study, we found that low visual fusion range is a risk factor for ACL sports injury. When one fixes one’s eyes on an object, two slightly different images are formed in one’s retina and then are merged into one image in the cerebral cortex. The range of visual fusion can reflect this ability. It belongs to the second level of stereo visual function [11,12,13]. The larger the range of stereo vision fusion, the better the stereo vision fusion function [14].

We did not find a direct correlation between abnormal vision and ACL sports injury. At present, there is no literature to support that abnormal vision increases the risk of ACL sports injury. Vision and visual fusion range are not the same, but they are related. Some scholars believe that this is due to anisometropia [11,12,13]. This study also indicates that fusion range is closely related to ametropia. People with abnormal vision (ametropia) are more likely to have abnormal stereo vision fusion range (87.6%). Therefore, in this study, we used multivariate logistic regression analysis to eliminate the confounding factors caused by the correlation between items as much as possible.

Questionnaires and visual examination were performed to establish whether participants had strabismus. The statistical significance of the two results was inconsistent, which may be due to the fact that the questionnaire data were biased by the subjective thinking of the participants. Therefore, it cannot be concluded that strabismus is a risk factor for ACL injury.

For the site factors, we found that hard sites, such as cement floor and wood floor, are more likely to cause ACL sports injury, which is consistent with previous studies [15,16,17]. Explained from the mechanism of ACL injury, it is actually the function of ground reactive force disturbances, that is, the loading effect of sheer force on knee joint [18], and sheer force is an important risk factor for knee motion injury, especially non-contact injury of ACL [19].

For weather factors, we found that the athletes with low visual fusion range have the highest risk of ACL injury in the cloudy weather. We believe that this is because insufficient light on cloudy days increases the negative impact of abnormal fusion on proprioception balance. 

For the injury action, our results showed that the action during injury is an important risk factor affecting ACL injury. The OR value of quick turn is the highest, which is consistent with the previous biomechanical studies that have shown that the actions that cause the maximum load of ACL in sports often include rapid change of direction with knee torsion [20,21,22,23]. In addition, the factors that may lead to the possibility and severity of ACL injury also include the stress and torque borne by the body, the body posture during landing, the completion of technical actions and the ground after jumping [19]. Therefore, the injury action can, indeed, affect the risk of ACL injury to a great extent. Sports that often involve such high-risk actions include football, basketball and skiing, which are also the sports with the highest incidence of ACL sports injury [24,25].

The results of this study provide a theoretical basis for the correlation between visual function and ACL injury risk and provide some sports protection guidance for athletes. Based on the conclusions of this study, we suggest that athletes, especially those with visual function defects, choose sports venues with sufficient light and soft ground, such as grass and artificial turf, and avoid fierce knee torsion. Meanwhile, this study can help sports lovers to pay more attention to the screening of stereo visual function. For the people who have been found to have stereo visual function defects, especially relating to the visual fusion range, we suggest that they should carry out standardized and targeted visual function training as soon as possible to restore stereoscopic visual function [26,27,28].

## 5. Conclusions

We found that abnormal visual fusion range is a risk factor for ACL injury. Athletes with abnormal visual fusion have increased risk of ACL non-contact and contact sports injury when they make a quick turn, sudden stop or jump on hard ground, such as wood floor, plastic ground or cement ground, on cloudy days.

## Figures and Tables

**Table 1:** Statistical description of 287 knee injury questionnaires.

	ACL-I	ACL-D	T (χ^2^)	*p*-Value
Total	133 (46.34%)	154 (53.66%)		
Sex				0.095
Male	95 (71.43%)	123 (79.87%)	2.785	
Female	38 (28.57%)	31 (20.13%)		
Age (year)	30.10 ± 10.24	28.31 ± 9.49	1.538	0.288
Weight (kg)	66.13 ± 13.21	69.39 ± 12.14	−2.173	0.464
Body Mass Index (kg/m^2^)	22.76 ± 3.24	23.46 ± 2.89	−1.936	0.222
Injury Side			0.001	0.976
Dominant	84 (63.16%)	97 (62.99%)		
Non-Dominant	49 (36.84%)	57 (37.01%)		
Injury Action				0.010 *
Sprint	22 (16.54%)	17 (11.04%)	15.147	
Quick Turn	39 (29.32%)	55 (35.71%)		
Sudden Stop	5 (3.76%)	17 (11.04%)		
Jump	32 (24.06%)	45 (29.22%)		
Retrogress	19 (14.29%)	9 (5.84%)		
Others ^#^	16 (12.03%)	11 (7.14%)		
Contact Confrontation			0.146	0.702
Yes	37 (27.82%)	46 (29.87%)		
No	96 (72.18%)	108 (70.13%)		
Site Condition			8.615	
Cement Floor	50 (38.59%)	63 (40.91%)		
Artificial Turf	21 (15.79%)	25 (16.23%)		0.125
Wooden Floor	10 (7.52%)	15 (9.74%)		
Plastic Floor	20 (15.04%)	33 (21.43%)		
Real Turf	4 (3.01%)	3 (1.95%)		
Others ^##^	28 (21.05%)	15 (9.74%)		
Weather			0.965	0.915
Shine	109 (81.95%)	129 (83.77%)		
Cloud	10 (7.52%)	13 (8.44%)		
Rain	6 (4.51%)	5 (3.25%)		
Snow	6 (4.51%)	6 (3.90%)		
Others ^###^	2 (1.50%)	1 (0.65%)		
Vision Right			4.130	0.389
Normal	42 (31.58%)	57 (37.01%)		
Low Ametropia	35 (26.32%)	43 (27.92%)		
Moderate Ametropia	43 (32.33%)	46 (29.87%)		
High Ametropia	11 (8.27%)	5 (3.25%)		
Unclear	2 (1.5%)	3 (1.95%)		
Vision Left			5.208	0.391
Normal	42 (32.58%)	61 (39.61%)		
Low Ametropia	38 (28.57%)	42 (27.27%)		
Moderate Ametropia	42 (31.58%)	43 (27.92%)		
High Ametropia	8 (6.02%)	4 (2.60%)		
Unclear	3 (2.26%)	4 (2.60%)		
Eye Diseases			8.703	0.069
Normal	84 (63.16%)	79 (51.30%)		
Astigmatism	40 (30.08%)	58 (37.66%)		
Amblyopia	1 (0.75%)	3 (1.95%)		
Strabismus	2 (1.50%)	10 (6.49%)		
Multiple Diseases	6 (4.51%)	4 (2.60%)		
Repeated Injuries			1.022	0.312
Yes	45 (33.83%)	61 (39.61%)		
No	88 (66.17%)	93 (60.39%)		

* means the item has statistical significance, *p* < 0.05. ^#^ mainly includes squat thrust, kick, pedal, etc. ^##^ mainly includes swimming pool, beach, dirt, etc. ^###^ mainly includes hail, windy, foggy, etc.

**Table 2:** Results of univariate logistic regression analysis of 287 knee injury questionnaires.

	Chi-Square Statistic	*p*-Value
Age	2.310	0.129 ǂ
Sex	2.763	0.096 ǂ
Weight	4.558	0.033 *
BMI	3.670	0.055 ǂ
Injury Side	0.001	0.976
Vision Right	3.901	0.420
Vision Left	3.916	0.562
Eye Diseases	7.912	0.095 ǂ
Combat	0.146	0.702
Injury Action	14.292	0.014 *
Site Condition	8.296	0.141 ǂ
Weather	0.934	0.920
Repeated Injuries	1.021	0.312

ǂ means the item has statistical significance, *p* < 0.2; * means the item has statistical significance, *p* < 0.05.

**Table 3:** Results of multivariate logistic regression analysis of 287 knee injury questionnaires.

	Estimate	SE	OR	95% CI		*p*-Value
Intercept	−0.542	0.352	0.581	0.286	1.149	0.123
Eye Diseases (Astigmatism)	0.477	0.270	1.611	0.951	2.751	0.078
Eye Diseases (Amblyopia)	1.344	1.189	3.832	0.458	80.582	0.258
Eye Diseases (Strabismus)	1.699	0.806	5.468	1.340	37.066	0.035 *
Eye Diseases (Multiple Diseases)	−0.438	0.714	0.646	0.146	2.589	0.540
Injured Action (Quick Turn)	0.687	0.396	1.989	0.922	4.377	0.082
Injured Action (Sudden Stop)	1.645	0.620	5.183	1.623	19.021	0.008 *
Injured Action (Jump)	0.608	0.408	1.837	0.830	4.139	0.136
Injured Action (Retrogress)	−0.447	0.535	0.640	0.218	1.805	0.404
Injured Action (Others)	0.034	0.523	1.035	0.367	2.886	0.948

* means the item has statistical significance, *p* < 0.05.

**Table 4:** Statistical description of 377 visual examination data.

	ACL-I	ACL-D	Control	F (χ^2^)	*p*-Value
Total (*n*, %)	127 (33.69%)	145 (38.46%)	105 (27.85%)	1.412	0.494
Male	92 (72.44%)	114 (78.62%)	79 (75.24%)		
Female	35 (27.56%)	31 (21.38%)	26 (24.76%)		
Age (year)	28.72 ± 8.81	27.71 ± 8.23	27.61 ± 7.99	0.617	0.512
Dominant eye				0.248	0.883
Right	106 (83.46%)	124 (85.52%)	88 (83.81%)		
Left	21 (16.54%)	21 (14.48%)	17 (16.19%)		
Pupillary Distance	62.06 ± 2.71	62.31 ± 2.76	63.23 ± 10.02	1.278	0.280
Vision Right	0.74 ± 0.45	0.73 ± 0.50	0.75 ± 0.39	0.035	0.966
≥1.0	60 (47.24%)	63 (43.45%)	47 (44.76%)		
0.1~1.0	57 (44.88%)	69 (47.59%)	49 (46.67%)		
≤0.01	10 (7.87%)	13 (8.97%)	9 (8.57%)		
Vision Left	0.75 ± 0.46	0.77 ± 0.50	0.75 ± 0.36	0.082	0.922
≥1.0	59 (46.46%)	68 (46.90%)	46 (43.81%)		
0.1~1.0	58 (45.67%)	66 (45.52%)	52 (49.52%)		
≤0.01	10 (7.87%)	11 (7.59%)	7 (6.67%)		
Oblique Perspective (°)	1.97 ± 1.91	1.99 ± 2.19	1.69 ± 1.88	0.770	0.464
≥5	12 (9.45%)	23 (15.86%)	10 (9.52%)		
<5	115 (90.55%)	122 (84.14%)	95 (90.48%)		
Fusion Range (°)	20.26 ± 7.27	17.82 ± 6.17	22.07 ± 5.16	14.103	<0.001 **
≥25	38 (29.92%)	29 (20.00%)	30 (28.57%)		
<25	89 (70.08%)	116 (80.00%)	75 (71.43%)		
Stereoacuity (″)	221.89 ± 411.15	273.93 ± 522.72	176.76 ± 361.42	1.464	0.233
≤60	87 (68.50%)	97 (66.90%)	78 (74.29%)		
60″~800	34 (26.77%)	37 (25.52%)	24 (22.86%)		
>800	6 (4.72%)	11 (7.59%)	3 (2.86%)		

** means the item has statistical significance, *p* < 0.01.

**Table 5:** Results of univariate logistic regression analysis of 377 visual examination results.

	Chi-Square Statistic	*p*-Value
Sex	1.415	0.493
Age	1.344	0.511
Dominant Eye	2.600	0.272
Pupillary Distance	0.250	0.882
Vision Right	0.070	0.965
Vision Left	0.165	0.921
Oblique Perspective	1.596	0.450
Fusion Range	27.377	<0.001 **
Stereoacuity	3.026	0.220

** means the item has statistical significance, *p* < 0.01.

**Table 6:** Results of multivariate logistic regression analysis of 377 visual examination results.

	Estimate Value	SE	OR	95% CI		*p*-Value
ACL-I to Control	1.070	0.450	2.915	1.207	7.038	0.017 *
ACL-I to Control	2.417	0.449	11.213	4.654	27.014	<0.001 **
ACL-I to ACL-D	−0.042	0.020	0.959	0.922	0.998	0.040 *
ACL-I to ACL-D	−0.106	0.021	0.900	0.863	0.938	<0.001 **

* means the item has statistical significance, *p* < 0.05; ** means the item has statistical significance, *p* < 0.01.

**Table 7:** Results of multivariate logistic regression analysis of combined data.

	Estimate Value	SE	OR	95% CI		*p*-Value
Fusion Range	−0.059	0.019	0.943	0.907	0.979	0.003 **
Action (Sprint)	0.692	0.486	2.000	0.768	5.213	0.155
Action (Quick Turn)	1.413	0.421	4.107	1.829	9.555	0.001 **
Action (Sudden Stop)	2.478	0.692	11.914	3.317	52.010	<0.001 **
Action (Jump)	1.500	0.454	4.483	1.876	11.195	0.001 **
Action (Retrogress)	0.321	0.557	1.378	0.451	4.072	0.565
Action (Others)	0.616	0.581	1.852	0.583	5.792	0.289

** means the item has statistical significance, *p* < 0.01.

**Table 8:** Odd ratio (OR) value of combined risk factors (non-contact injury).

		Sudden Stop	Jump	Quick Tun
Cloud	Wooden floor	13.208	4.439	3.482
	Plastic floor	11.880	3.993	3.132
	Cement floor	11.872	3.990	3.130
Snow	Wooden floor	12.962	4.356	3.417
	Plastic floor	11.659	3.918	3.073
	Cement floor	11.651	3.916	3.071
Rain	Wooden floor	11.782	3.960	3.106
	Plastic floor	10.598	3.562	2.794
	Cement floor	10.591	3.560	2.792

**Table 9:** Odd ratio (OR) value of combined risk factors (contact injury).

		Sudden Stop	Jump	Quick Tun
Cloud	Wooden floor	10.583	3.557	2.790
	Plastic floor	9.519	3.199	2.509
	Cement floor	9.513	3.197	2.508
Snow	Wooden floor	10.386	3.491	2.738
	Plastic floor	9.342	3.140	2.463
	Cement floor	9.336	3.138	2.461
Rain	Wooden floor	9.441	3.173	2.489
	Plastic floor	8.492	2.854	2.239
	Cement floor	8.486	2.852	2.237

## Data Availability

Not applicable.
